# Troubled Topolobampo

**Published:** 1890-03

**Authors:** S. H. Preston


### TROUBLED TOPOLOBAMPO.

BY S. H. PRESTON.

The tenacity with which Dr. Babbitt still sticks to Topolobampo, in spite of the repeated and recent exposures of people who have been there and succeeded in getting back to the sinful and competitive world, is surprising. Facts seem to have no effect upon his faith in "Pacific City," a city still unfounded, save in the fancy of its infatuated and impractical projectors. Verily, he betrays an unbounded belief in the verity of "things not seen."

Dr. Babbitt charges me with befogging and misrepresenting. I challenge him to specify a single statement not strictly true.

The proposed site of "Pacific City" is now occupied by about one hundred people housed in tattered tents, trying the best they can to keep their souls in their bodies with corn-bread and black-eyed beans. And this after fourteen years of utopia building upon a foreign coast.

As previously remarked, the oldest residents are those unable to abscond. All who do get away tell the same tale of disappointment and privation. Time and time again has the press punctured this co-operative bubble. That even a few folks have been lured to Topolobampo, is due to the dereliction of an efficient fool-killer who has to deal with a population of nearly 100,000,000.

Since the late thorough exposure by our city dailies, another "integral co-operationist" has had the luck to again reach our competitive country, and reports through the Chicago *Tribune:*

"The Socialistic colony at Topolobampo, Mexico, seems to be in a bad way. One C. C. Remley, who went from Kansas a year ago to join the society and get relief from the tyranny of capital, has made his way back and reports that the colony is composed of 'old men and women who have become so embittered in their disappointment that they are hard to live with.' They would leave if they could get away, but 'having put their money in the general fund, they can get nothing back,'

and 'are paid for work in time checks which cannot be cashed, because the directors claim there are no funds.' Remley says 'the regular diet of the colonists consists of black-eyed beans and corn-bread three times a day, as most of them are too poor to buy any meat.'"

It is to be presumed that Mr. Remley, who has been to Topolobampo, knows as much about it as any man who has not been there. It will not do for the managers of this Mexican enterprise to denounce all the absconders, who corroborate each other in their accounts, as disaffected drones or deliberate liars. In the competitive and unco-operative world, personal knowledge and experience count for more than the bald assertions of blinded or interested absentees.

I called the encampment of torn tents at Topolobampo a community. With this Dr. B. finds fault. If I call it a colony he objects, because Botany Bay is a colony. I cannot please him any way. But I will bet that the bill of fare is better and the folks better furnished with all the comforts of life at the latter colony than at the former. It seems they get away from both colonies when they can. But the Botany Bay establishment has this advantage over the Mexican concern—it has a backing, the backing of the British Government—and it has facilities for forcing its refractory members to remain.

Instead of admitting the destitution and distress at Topolobampo, the Doctor dwells upon the beauty of the climate and such matters. The poor people would probably prefer less climate and more meat.

The Doctor states that "some of the noblest and purest souls that walk this earth are members of that colony." It matters not though all the ransomed souls from Maine to Mexico were members, with Gabriel for governor, "under the laws of Colorado," it would not change the actual condition of the colony to-day at Topolobampo.

It is strange that so many Remleys run away from this rendezvous of pure and noble souls. The Doctor declares the colonists are the "choice people of the earth," and are "contented and harmonious." It is queer that folks will quit this earthly Eden for the capital-cursed and competitive world. I fear the Doctor is "befogging."

---

Forty-five of the Presbyteries of the Presbyterian church, representing 1,697 ministers and 251,236 communicants, have voted to revise the Westminister standards. The vote stands 32 in favor of revision and 12 against.

